# Detecting five-pattern personality traits using eye movement features for observing emotional faces

**DOI:** 10.3389/fpsyg.2024.1397340

**Published:** 2024-09-24

**Authors:** Ying Yu, Qingya Lu, Xinyue Wu, Zefeng Wang, Chenggang Zhang, Xuanmei Wu, Cong Yan

**Affiliations:** ^1^School of Life Sciences, Beijing University of Chinese Medicine, Beijing, China; ^2^College of Information Engineering, Huzhou University, Huzhou, China

**Keywords:** eye tracking, five-pattern personality traits, machine learning, emotional faces, personality prediction

## Abstract

The five-pattern personality traits rooted in the theory of traditional Chinese medicine (TCM) have promising prospects for clinical application. However, they are currently assessed using a self-report scale, which may have certain limitations. Eye tracking technology, with its non-intrusive, objective, and culturally neutral characteristics, has become a powerful tool for revealing individual cognitive and emotional processes. Therefore, applying this technology for personality assessment is a promising approach. In this study, participants observed five emotional faces (anger, happy, calm, sad, and fear) selected from the Chinese Facial Affective Picture System. Utilizing artificial intelligence algorithms, we evaluated the feasibility of automatically identifying different traits of the five-pattern personality traits from participants’ eye movement patterns. Based on the analysis of five supervised learning algorithms, we draw the following conclusions: The Lasso feature selection method and Logistic Regression achieve the highest prediction accuracy for most of the traits (TYa, SYa, SYi, TYi). This study develops a framework for predicting five-pattern personality traits using eye movement behavior, offering a novel approach for personality assessment in TCM.

## 1 Introduction

Personality traits, as relatively enduring and stable patterns of thought, feeling, and behavior, convey important information about individuals. A broad definition of personality encompasses a set of behavioral, cognitive, and emotional patterns that predict an individual’s behavior and interaction with the environment (Loehlin, 1992). Internationally, Eysenck’s personality theory has been instrumental in elucidating the neurophysiological causes of an individual’s personality (Mitchell and Kumari, 2016; Kumari et al., 2004), and the Big Five personality framework facilitates career advice, fostering social relationships, and identifying mental health problems (Li Y. et al., 2015; Yu et al., 2021; Waltz and Chou, 2023). Each framework offers its lens through which to perceive the intricacies of human personality, however, some scholars still debate whether these personality theories can objectively reflect personality traits independently of cultural influences. Therefore, many psychologists have been developing indigenous personality theories rooted in specific cultural experiences, such as those in Japan, Korea, and India (Cheung et al., 2003; Fruyt et al., 2015). In China, the five-pattern personality theory, rooted in traditional Chinese medicine, is considered the most culturally distinctive, and it is widely used in clinical settings (Qie and Wu, 2022). The differences in the balance of yin and yang within the human body not only lead to diverse physiological characteristics but also reflect a range of individual personality and behavioral traits on a deeper level.

The study of five-pattern personality traits relies primarily on the five-pattern personality inventory, which consists of six subscales: TYa, SYa, Yy, SYi, TYi, and a masking scale, with a total of 103 questions. Research on the five-pattern personality traits using this inventory currently focuses on three main areas. First, researchers examine the differences in subscale scores between patient groups and healthy individuals. Current studies on the relationship between the five-pattern personality traits and diseases primarily focus on chronic illnesses, gynecological conditions, and mental health issues (Zheng et al., 2007; Wang et al., 2023). Second, extensive research explores the correlation between subscale scores and various issues, such as career anxiety, depression, and internet addiction (Zhang et al., 2013; Chen et al., 2021). Lastly, studies classify individuals into different groups based on their subscale scores to explore the physiological differences in the five-pattern personality traits from a modern neurobiological perspective (Li et al., 2020; Zhao et al., 2020).

Currently, assessing personality traits primarily relies on self-report measures, where participants respond to a series of statements or adjectives, making retrospective judgments about their own personality. However, scales relying solely on personal recollection may be influenced by the participants’ subjective awareness and societal expectations, potentially biasing the results. Advances in information technology offers potential solutions to these challenges. As an advanced research method, eye tracking technology provides real-time data by measuring uncontrollable physiological response signals and is widely used in the fields of psychology and behavioral science (Abadi et al., 2015; Miranda-Correa et al., 2021). For instance, eye tracking technology has been used to detect stress by measuring pupil diameter (PD), which increases under stress due to activation of the sympathetic nervous system (Yousefi et al., 2022). Another application is cognitive load assessment, where changes in PD reflect the mental effort required to complete tasks (Abdi Sargezeh et al., 2019). Eye tracking technology has expanded the cognitive boundaries of individual visual behavior. It allows researchers to analyze eye movement patterns in detail, gaining insights into information processing, decision-making, and differences in attention allocation to external stimuli.

Eye tracking technology can identify emotions and analyze how individuals view specific images, videos, or facial expressions (Wieckowski and White, 2017; Lim et al., 2020). Eye tracking plays a crucial role in the decision-making. Studies have shown that personality differences significantly affect decision-making styles, including risk-taking tendencies and preferences for movies, music, and books (Nicholson et al., 2005; Pachur and Spaar, 2015; Wu et al., 2018).

Eye-tracking data can also infer individual personality traits. Compared to traditional self-report methods, it offers a more objective assessment by bypassing the language barriers and subjective biases associated with personality measurement scales (Koutsogiorgi and Michaelides, 2022; Chen et al., 2023). Moreover, portable eye-tracking devices are simple to operate and easy to transport, facilitating personality assessment in natural environments and aiding the development of assessments in daily life contexts (Hoppe et al., 2018).

Therefore, this study combines eye tracking technology with artificial intelligence algorithms to develop a model for predicting the five-pattern personality traits based on eye movement features. In practical terms, standard emotional facial expressions were presented to the participants, and the relevant eye movement data were processed using the Mutual information and Lasso feature selection methods. Subsequently, we employed five machine learning models—Decision Tree (DT), K-Nearest Neighbor (KNN), Logistic Regression (LR), Naive Bayes (NB), and Support Vector Machine (SVM)—to identify five-pattern personality traits, with LR yielding the optimal predictive results.

## 2 Materials and methods

### 2.1 Personality assessments

According to the differences between Yin and Yang, the five-pattern personality theory of TCM divides individuals into five traits: TYa, SYa, Yy, SYi, and TYi. Table 1 briefly introduces the characteristics of different personalities (Yang and Xue, 2006). To make the model more universal, the participants in this study were individuals without psychological or personality problems. Based on the results of previous studies and the distribution of participants, we divide the true values obtained in the personality inventory into three levels: low, medium, and high. We then discretized these scores to be used as category labels of eye movement signals. This enables us to express the task of personality prediction as a classification problem and use discrete class labels for training and prediction.

**Table 1:** Summary of the studied personality traits.

Trait	Description	Group		
		Low	Medium	High
TYa	Reflect the intensity. People with high TYa scores are arrogant, self-employed, subjective, impulsive, ambitious, spirited, capricious without regard to right and wrong, irritable, stalwart and courageous, impassioned, enterprising, dare to insist on one’s own point of view, etc.	[0, 10]	[11, 15]	[16, 20]
SYa	Reflects flexibility. People with high SYa scores are sociable, cheerful, agile and optimistic, frivolous and volatile, witty, easy-going, careless, like to talk and laugh, do not want to be quiet but want to be active, have lots of friends, enjoy recreational activities, are not easy to stick to things, etc.	[0, 9]	[10, 15]	[16, 22]
Yy	Reflects balance. People with high Yy scores have a composed demeanor, dignity and modesty, joy and anger do not manifest in their expression, quiet living, are not influenced or swayed by external things, have no selflessness and fearlessness, no worries, no complacency, can conform to the development law of things, etc.	[0, 4]	[5, 7]	[8, 10]
SYi	Reflects persistence. People with high SYi scores are cold and calm, good at recognizing right and wrong, deeply contemplative without outward display, can exercise self-control, have a plan for doing things, do not speak randomly, do not act rashly, are cautious, careful, steady, vigilance, jealous, soft and weak, have the ability to last, etc.	[0, 11]	[12, 16]	[17, 21]
TYi	Reflects convergence. People with high TYi scores are modest, thoughtful, pessimistic, timid, indecisive, keep a certain distance from people, introspective and lonely, unwilling to interact with others, conservative, selfish, basing one’s actions on others’ success or failure, unwilling to take the lead or initiative, etc.	[0, 7]	[7, 13]	[14, 22]

The inventory used in this study is a fully researched and verified tool, which has been used in a series of personality testing tasks in personality research (Li L. et al., 2015). This study was approved by the Ethics Committee of Beijing University of Chinese Medicine (2022BZYLL05011). All participants agreed to the experiment and provided signed informed consent.

### 2.2 Apparatus

The eye movement behavior of the participants was recorded using a Tobii 4C eye tracker, and the stimulation was presented by a standard 23.8-inch LCD with a screen resolution of 1,920 × 1,080 pixels. The participants sat in a chair with their heads on a fixed chin rest, keeping their eyes about 70 cm away from the monitor. In addition, to ensure that the eye tracker could accurately track the participants’ fixation points, all participants underwent a standard 9-point calibration and verification test before the experiment officially started. The data were collected in a controlled laboratory environment with fixed illumination and room temperature, and each participant was collected individually.

### 2.3 Procedure

**Figure 1:**
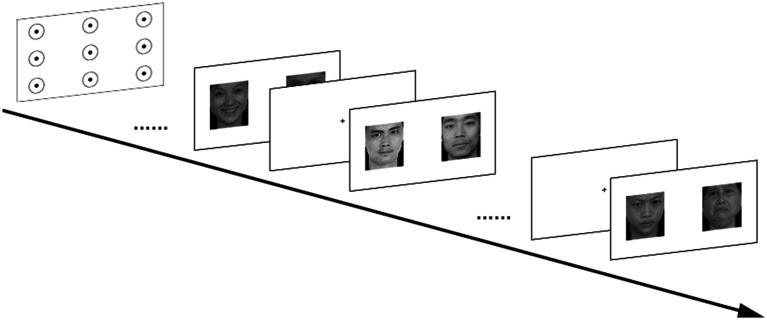
Process of observing emotional face pictures. The emotional face images were all obtained from the Chinese Facial Affective Picture System (Gong et al., 2011).

In this study, static image emotional stimulation was used to arouse the emotional response of the participants. The eye movement behavior of the participants was recorded when they freely observed each emotional face combination image (Figure 1). The Chinese Facial Affective Picture System (Gong et al., 2011) was used in the experiment. Based on the valence and arousal of different emotions, pictures that scored higher for five emotional faces (anger, happy, calm, sad, and fear) were selected, with a total of 20 pictures, 4 for each emotion, maintaining a 1:1 male-to-female ratio. The images were combined in pairs according to the same gender and different emotions, and 10 emotional face combination images were presented to all participants in the same order. The display time of each combined image was set to 5 s, and a 1 s black cross picture appeared between two images to ensure that the original gaze point of each image started from the center.

### 2.4 Eye movement features

The features extracted from the recorded eye movement data are related to eye activity and can be categorized into three types: complex eye movement patterns, eye-movement Shannon entropy and fixation duration of specific area of interest. Complex eye movement patterns describe eye movement information through physiological objective indicators (Holland and Komogortsev, 2011), capturing overall patterns and behavioral characteristics of eye movements. Eye-movement Shannon entropy is a key indicator of gaze dispersion, assessing the complexity and uncertainty in eye movement data from the perspective of information entropy (Wang et al., 2020). Fixation on specific parts of the face, such as the eyes, nose, and mouth, can convey different meanings related to emotions, personality, identity, and communication (Schurgin et al., 2014). Fixation duration in these areas is an important metric for evaluating visual attention allocation, reflecting the time spent focusing on specific regions, which provides insights into an individual’s attention strategy in visual tasks.

Complex eye movement patterns include 14 indicators, which are fixation count, mean fixation duration, mean vectorial saccade amplitude, mean horizontal saccade amplitude, mean vertical saccade amplitude, mean vectorial saccade velocity, mean vectorial saccade peak velocity, velocity waveform indicator, scanpath length, scanpath convex hull area, regions of interest, inflection count, slope of the amplitude-duration relationship, and slope of the main sequence relationship. They can be used to objectively evaluate the biological characteristics of eye movement and their ability to accurately and precisely distinguish between unique individuals. The raw eye movement data were processed by MATLAB R2016a, and finally 140 complex eye movement pattern-related features were included.

Eye-movement Shannon entropy is used to measure the statistical randomness or aggregation of the participants’ eye movements. Spatially dispersed gaze leads to higher entropy values, while spatially tightly focused gaze leads to lower entropy values. MATLAB R2016a was used to draw the duration heatmap for each valid trial of each participant, and then Shannon entropy was calculated on the heatmap, and finally 10 related features were included.

For the combination of emotional face pictures, participants can point their visual attention to a face or one of the three face elements (eyes, nose and mouth), thus showing their visual attention preference. First, the 10 combined images were divided into regions of interest using Adobe Photoshop CC 2019 with a single emotional face picture, the eyes, nose, and mouth portions of the different face pictures. Next, the matplotlib and cv2 libraries in Python 3.9 were used to label the fixation points recorded by self-developed eye movement data software, and to calculate the total fixation duration in different interest areas for each image, resulting in 110 related features.

### 2.5 Feature selection

Traditionally, feature selection algorithms are divided into three main categories: filter, wrapper and embedded methods. Filter methods are independent of specific learning algorithms and rank the importance of features using a particular evaluation function, selecting the optimal feature subset. This method is simple, fast, and computationally efficient, making it very popular in practical applications. Mutual information (MI) measures the degree of information shared between two variables from an information theory perspective (Cover and Thomas, 1991). It is used to select the features with the highest information content for classification tasks. Embedded methods, on the other hand, automatically select the most relevant features during model training based on feature importance. The Least Absolute Shrinkage and Selection Operator (Lasso) is a typical embedded technique introduced by Tibshirani (1996). Lasso reduces the variable set by constructing a first-order penalty function, thereby selecting features with predictive power. This method combines feature selection and model training, making it more efficient and precise during the feature selection process. In this study, we used mutual information and Lasso for feature selection, implemented through the Scikit-Learn library in Python 3.9.

### 2.6 Classifier evaluation

A separate classifier was trained for the prediction of each personality trait, such that we ended up with 5 classifiers. A training data point corresponds to a participant, including the value of the selected feature and the label assigned based on the discrete score of the personality trait. In the realm of machine learning, supervised learning is a crucial paradigm. The core idea behind supervised learning is to train a model using a labeled training dataset, enabling the model to learn the relationship between input features and output labels. This allows the model to accurately predict or classify new, unseen data (Dridi, 2021). Given the significance of supervised learning in machine learning and its excellent predictive and classification capabilities, this study employed five robust supervised algorithms to predict the classification results of the five-pattern personality traits: Decision Tree (DT), K-Nearest Neighbors (KNN), Logistic Regression (LR), Naive Bayes (NB), and Support Vector Machine (SVM). DT is easy to understand and interpret. KNN is suitable for handling small sample data. LR has fewer parameters, making it less prone to overfitting. NB offers high computational efficiency, and SVM excels in high-dimensional spaces. To evaluate the performance of these classifiers, we utilized five-fold cross-validation and adjusted the model parameters to avoid overfitting. The models were then assessed using accuracy, F1 score, and AUC.

## 3 Results

### 3.1 Participant

A total of 57 people were recruited to participate in the eye movement experiment with the completion of the five-pattern personality inventory. Among them, 4 participants were excluded for scoring less than 5 on the masking scale, 1 was excluded due to problems in completing the inventory, and 1 was excluded due to missing eye movement data. Finally, 51 people were included in the analysis. Table 2 shows the demographic data of the participants and the statistical descriptions of different personality traits.

**Table 2:** Descriptive statistics of participants.

Trait	Sex Male (female)	Age M (P25, P75)	Descriptive statistics			Shapiro–Wilk normality test	
			Range	Mean	SD	Statistic	*p* value
TYa	14 (37)	30 (24.5, 41)	[4, 17]	11.24	3.296	0.9635	0.1176
SYa			[5, 21]	13.06	3.495	0.9779	0.4545
Yy			[0, 10]	6.588	2.617	0.9365	0.0089
SYi			[6, 20]	13.08	3.649	0.9679	0.1811
TYi			[0, 19]	7.510	5.025	0.9418	0.0145

After performing the chi-square test, it was found that there were no significant differences in gender distribution across the five personality traits (TYa:χ^2^ = 1.097, *p* = 0.5779; SYa:χ^2^ = 1.048, *p* = 0.5920; Yy:χ^2^ = 3.527, *p* = 0.1715; SYi:χ^2^ = 4.656, *p* = 0.0975; TYi:χ^2^ = 3.398, *p* = 0.1829). Among the 51 participants included in this study, the minimum age was 19 and the maximum age was 59. The Kruskal-Wallis test also showed no statistically significant differences in age distribution across the five personality traits (TYa: H = 3.189, *p* = 0.2030; SYa: H = 0.3677, *p* = 0.8321; Yy: H = 3.341, *p* = 0.1881; SYi: H = 3.004, *p* = 0.2227; TYi: H = 1.896, *p* = 0.3874), indicating that the selected sample has good representativeness.

### 3.2 Feature selection methods

**Figure 2:**
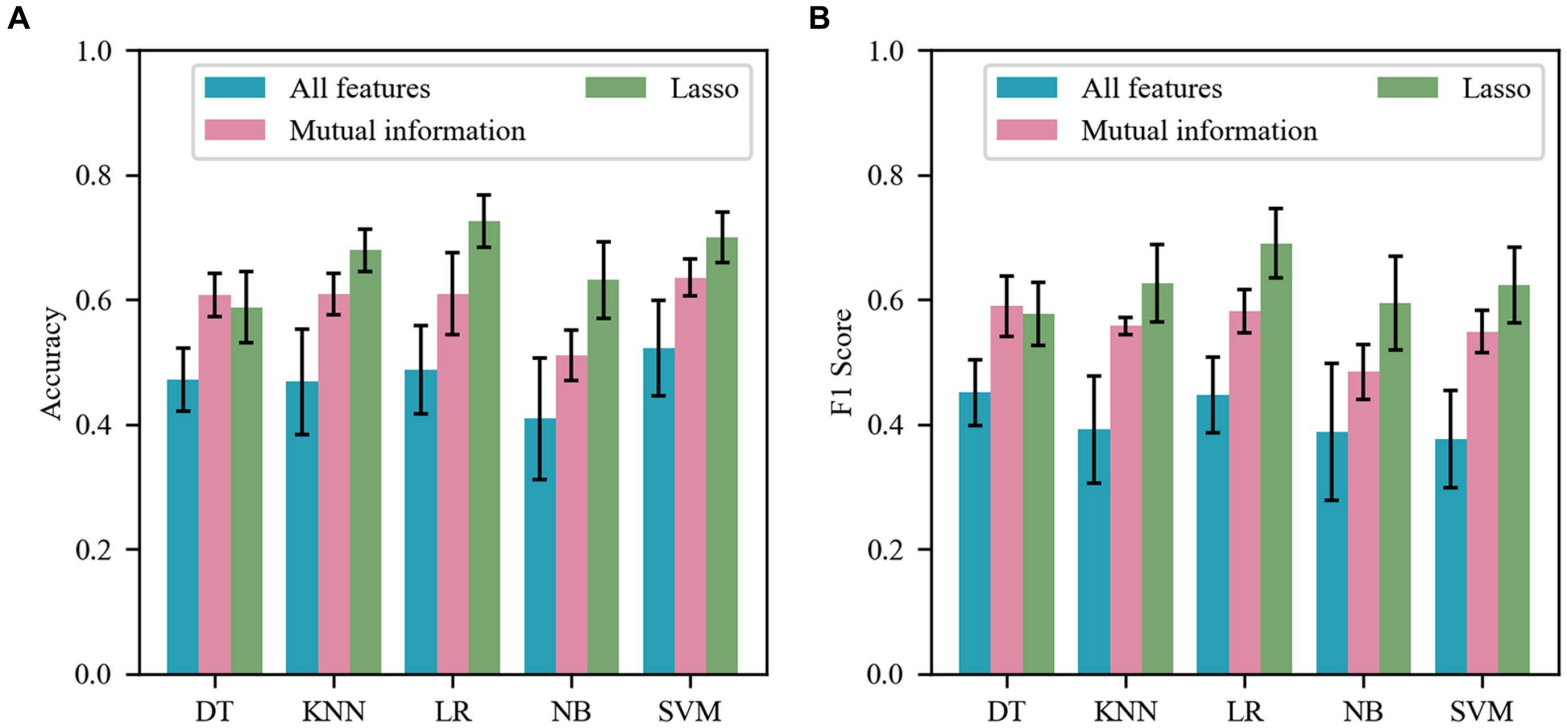
Comparison of feature selection. **(A)** Mean accuracy of the five classifiers for each personality trait at baseline and after feature selection. **(B)** Mean F1 Score of the five classifiers for each personality trait at baseline and after feature selection.

To evaluate the effect of feature selection method, we first fused the five trait-specific classifiers into one metric by averaging the prediction results over the five personality traits. We also compared it with the baseline setting that uses all 260 features without feature selection. Figure 2 shows the average accuracy and F1 Score of five classifiers and all baselines for each personality trait. As can be seen from the figure, classifiers with feature selection perform significantly better than those without feature selection. This indicates that feature selection is crucial for enhancing the accuracy of personality predictions. Specifically, both mutual information and Lasso methods improve prediction performance.

According to the results in Figure 2, Lasso demonstrated higher accuracy and F1 score across the KNN, LR, SVM, and NB, while the mutual information performed better with the DT. Friedman test revealed that there were statistically significant differences in prediction accuracy between the different feature selection methods (χ^2^ = 8.400, *p* = 0.0085). Further analysis using Dunn’s multiple comparisons test revealed significant differences in accuracy between the baseline method and the Lasso method (*p* = 0.0133). Additionally, one-way repeated measures ANOVA showed statistically significant differences in F1 scores between the different feature selection methods (*F* = 58.43, *p* < 0.0001). The post-hoc analysis with Bonferroni adjustment further identified significant differences between the baseline method and two of the feature selection methods (MI: *p* = 0.0003; Lasso: *p* < 0.0001). Overall, Lasso has indeed shown performance improvements across most classifiers, with the Lasso-LR combination achieving the best results on average across five traits.

### 3.3 Classifier performance

Table 3 shows the results of five classifiers using the Lasso feature selection method to predict five-pattern personality traits. LR achieved the best accuracy in the TYa, SYa, SYi, and TYi. For the Yy, NB and SVM outperformed LR. When considering individual traits, we observe that all traits were predicted with accuracy greater than 0.7. Averaging performance across all the classifiers, TYa had the highest accuracy and F1 Score. The statistical analysis, including a one-way repeated measures ANOVA with Geisser-Greenhouse (*F* = 9.814, *p* = 0.0058) and the Friedman Test (χ^2^ = 7.200, *p* = 0.1257), revealed significant differences in prediction accuracy and mean F1 scores between five classifiers. The *post hoc* analysis with a Bonferroni adjustment revealed that the accuracy of LR is significantly different from NB (DT: *p* = 0.0501; KNN: *p* = 1.000; NB: *p* = 0.0375; SVM: *p* = 1.000). Among the five traits, the average accuracy of LR was 2.62–13.78% higher than other, significantly surpassing the accuracy of other classifiers. Furthermore, the highest accuracy in Figure 2 was achieved through the Lasso feature selection method. Overall, we conclude that the combination of LR with Lasso feature selection predicts traits more accurately than other combinations, and we will focus on this combination in future experiments.

**Table 3:** Comparison of five classifiers for the five personality traits.

Classifier	Metric	TYa (95%CI)	SYa (95%CI)	Yy (95%CI)	SYi (95%CI)	TYi (95%CI)
DT	Accuracy	0.6673 (0.5524, 0.7821)	0.5509 (0.4642, 0.6376)	0.5673 (0.4636, 0.6710)	0.5273 (0.4073, 0.6472)	0.6273 (0.5545, 0.7001)
	F1 Score	0.6449 (0.5356, 0.7542)	0.5586 (0.4635, 0.6538)	0.5585 (0.4449, 0.6721)	0.5139 (0.4103, 0.6176)	0.6075 (0.5208, 0.6942)
	AUC	0.7094 (0.6159, 0.8029)	0.6068 (0.4933, 0.7203)	0.6705 (0.5892, 0.7519)	0.5846 (0.4997, 0.6696)	0.6982 (0.6643, 0.7321)
KNN	Accuracy	0.6855 (0.5403, 0.8306)	0.6673 (0.6264, 0.7081)	0.7109 (0.5797, 0.8421)	0.7055 (0.6426, 0.7683)	0.6273 (0.5133, 0.7412)
	F1 Score	0.6248 (0.4532, 0.7964)	0.5783 (0.5573, 0.5994)	0.6615 (0.5501, 0.7729)	0.7096 (0.6635, 0.7557)	0.5571 (0.4036, 0.7107)
	AUC	0.7788 (0.6198, 0.9378)	0.618 (0.4983, 0.7376)	0.7856 (0.6960, 0.8751)	0.7457 (0.6748, 0.8166)	0.7191 (0.6345, 0.8037)
LR	Accuracy	0.7818 (0.6363, 0.9273)	0.7455 (0.6137, 0.8772)	0.6709 (0.5034, 0.8384)	0.7255 (0.6875, 0.7634)	0.7055 (0.6426, 0.7683)
	F1 Score	0.7596 (0.6124, 0.9068)	0.7248 (0.5787, 0.8710)	0.6298 (0.4584, 0.8013)	0.6961 (0.6538, 0.7384)	0.6398 (0.5612, 0.7185)
	AUC	0.8667 (0.7908, 0.9425)	0.8426 (0.7449, 0.9403)	0.8328 (0.7411, 0.9245)	0.8321 (0.7775, 0.8867)	0.7609 (0.6320, 0.8898)
NB	Accuracy	0.7036 (0.5494, 0.8579)	0.6673 (0.5706, 0.7640)	0.5927 (0.4722, 0.7132)	0.6455 (0.4839, 0.8070)	0.5491 (0.3911, 0.7071)
	F1 Score	0.6745 (0.5149, 0.8341)	0.6155 (0.5458, 0.6853)	0.5721 (0.4638, 0.6804)	0.6341 (0.4670, 0.8012)	0.4765 (0.3206, 0.6324)
	AUC	0.7195 (0.5973, 0.8416)	0.6631 (0.5809, 0.7452)	0.7303 (0.6214, 0.8392)	0.7757 (0.6418, 0.9096)	0.6974 (0.5665, 0.8282)
SVM	Accuracy	0.7655 (0.7231, 0.8078)	0.6891 (0.5879, 0.7903)	0.7091 (0.6177, 0.8005)	0.6691 (0.5625, 0.7756)	0.6655 (0.6122, 0.7187)
	F1 Score	0.7255 (0.6893, 0.7616)	0.5821 (0.4352, 0.7291)	0.6303 (0.5454, 0.7151)	0.5879 (0.4593, 0.7166)	0.5904 (0.5193, 0.6615)
	AUC	0.8708 (0.8064, 0.9353)	0.8012 (0.7244, 0.8781)	0.7984 (0.7038, 0.8930)	0.8072 (0.7150, 0.8994)	0.7676 (0.6493, 0.8858)

### 3.4 Eye movement feature type

**Figure 3:**
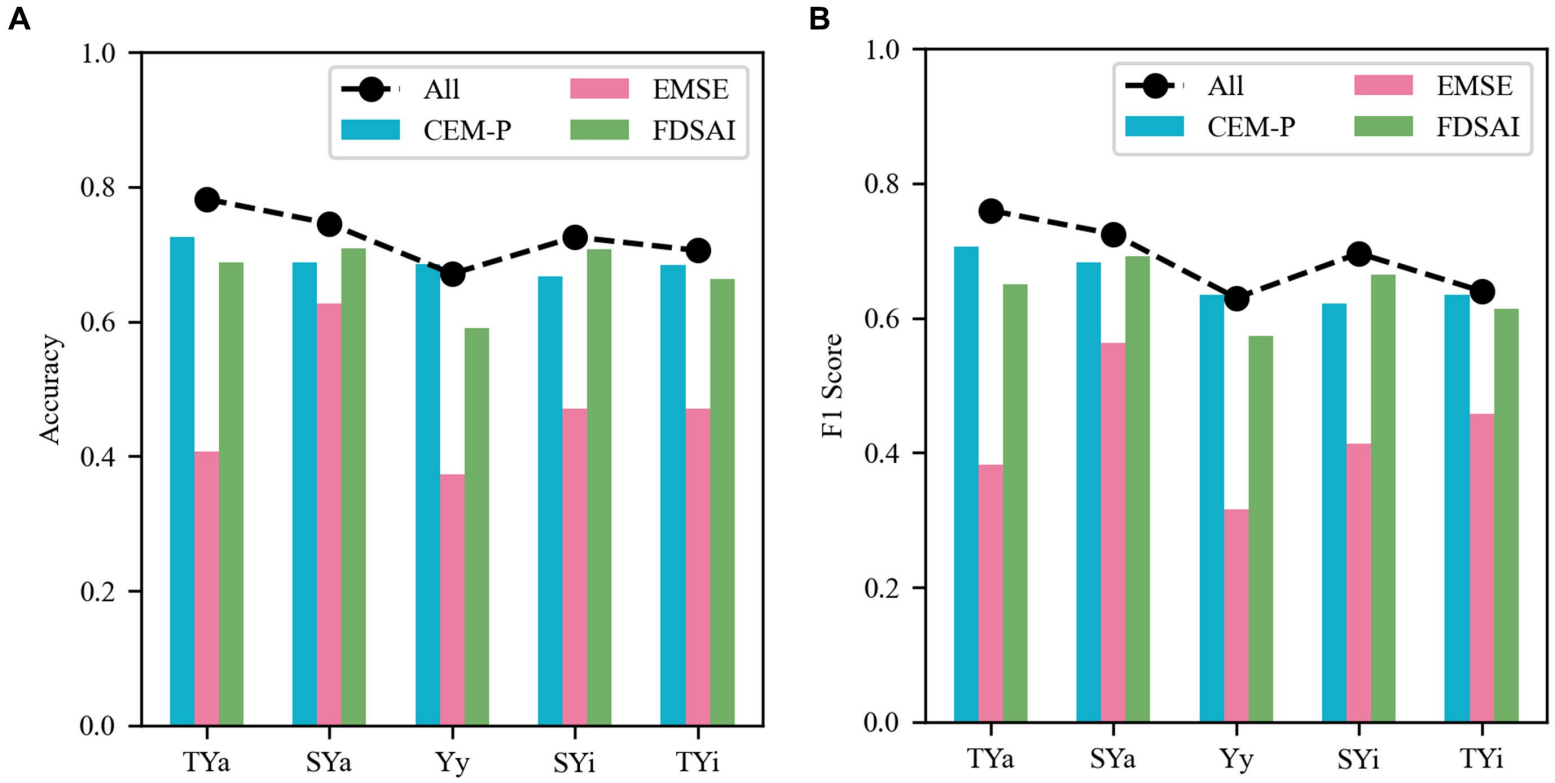
Personality trait prediction in different feature types. **(A)** Mean accuracy of three feature types and combined features for each personality trait. **(B)** Mean F1 Score of three feature types and combined features for each personality trait. Where CEM-P stands for Complex eye movement patterns, EMSE represents Eye-movement Shannon entropy, and FDSAI denotes fixation duration of specific area of interest.

In further analysis, we will determine which eye movement feature type—complex eye movement patterns, eye-movement Shannon entropy, or fixation duration of specific area of interest—is more informative for predicting personality traits. For this purpose, we divided the data into three groups and used the Lasso-LR model to train and predict different types of eye movement features. As shown in Figure 3, each type of eye movement features has its own strengths and weaknesses in predicting different traits. The average accuracy across all personality traits was 0.6898, 0.4698, and 0.6716, respectively, with average F1 scores of 0.6558, 0.4264, and 0.6385. The complex eye movement patterns outperformed the other two types in predicting TYa, Yy, and TYi, while the fixation duration of specific area of interest was more effective in evaluating SYa and SYi. On average, using complex eye movement patterns and the fixation duration of specific area of interest resulted in a prediction accuracy improvement of 22.00% and 20.18%, respectively. This difference may be due to more informative features are extracted from complex eye movement patterns and fixation duration of specific area of interest. Moreover, the entropy value solely employs Shannon entropy, which accounts for the distribution of gaze points, or the uncertainty of eyes resting on different observation targets, without introducing other metrics such as approximate entropy and fuzzy entropy (Harezlak and Kasprowski, 2020; Lee et al., 2022).

There are statistically significant differences in prediction accuracy and F1 scores among different feature types (Accuracy: *F* = 23.02, *p* = 0.0039; F1 Score: *F* = 34.92, *p* = 0.0015). The pairwise multiple comparisons revealed that the accuracy of eye-movement Shannon entropy is significantly different from complex eye movement patterns (*p* = 0.0286) and fixation duration of specific area of interest (*p* = 0.0111). Regarding F1 scores, statistically significant differences were observed between eye movement Shannon entropy and both complex eye movement patterns (*p* = 0.0135) and fixation duration of specific area of interest (*p* = 0.0055). While complex eye movement patterns and fixation duration of specific area of interest each demonstrated different strengths in predicting various personality dimensions, the differences between these two feature types were not statistically significant after Bonferroni correction (Accuracy: *p* > 0.9999; F1 Score: *p* > 0.9999). Earlier studies on personality have indicated that different eye movement features reflect various aspects of personality. For example, there is a significant correlation between BAS score and blink response (Taib et al., 2020). Individuals with high Neuroticism score show a significantly prolonged average gaze time and dwell time (Rauthmann et al., 2012), and Openness has a significant impact on eye movement parameters (Matsumoto et al., 2010; Wu et al., 2014). These findings emphasize the importance of carefully selecting eye movement features for predicting personality, as different feature combinations offer a more comprehensive understanding of individuals.

## 4 Discussion

In this study, we developed a framework based on the response of eye movements to emotional face stimulation to predict five-pattern personality traits. We found that the prediction effect of combining Lasso feature selection with Logistic regression was much better than other machine learning algorithms. Overall, the prediction accuracy ranged from 0.67 to 0.78, with F1 Scores all higher than 0.62. This result is quite similar to the previous literature reports on predicting Big Five personality. For example, when observing product information on a recommendation interface, the combination of the Gini coefficient and AdaBoost achieved an accuracy of 0.66 to 0.73 in predicting the Big Five personality traits (Chen et al., 2023). In a study where eye movement data were collected while users viewed different colors, fonts, and text arrangements, the prediction accuracy for all Big Five dimensions exceeded 0.79 (Al-Samarraie et al., 2018). However, in real-world scenarios, the classification F1 scores for the Big Five personality traits using wearable eye-tracking devices ranged from 0.3 to 0.5 (Hoppe et al., 2018).

In contrast, the combination of Lasso feature selection with the DT, KNN, NB, and SVM classifiers showed mediocre performance in predictive outcomes compared to the Lasso-Logistic model. After Lasso feature selection, the prediction accuracy of DT, KNN, LR, NB, and SVM increased by 0.1160, 0.2109, 0.2378, 0.2225, and 0.1771 on average, and the F1 Score increased by 0.1257, 0.2340, 0.2426, 0.2061, and 0.2464 on average. This difference may be due to the different adaptability of different machine learning models to data. Lasso achieves feature selection through L1 regularization, compressing coefficients of unimportant features to zero and thus eliminating them. For linear models like LR, the feature selection effect of Lasso may be more pronounced as they heavily rely on the quality of features for optimal performance (Qu et al., 2022).

In terms of specific individual personality traits, this study achieved optimal predictive results for the TYa. This outcome may be associated with neurobiological factors within the brain. Resting-state fMRI measurements revealed that individuals with high TYa scores exhibited higher regional homogeneity in the right superior temporal gyrus (STG), indicating more homogeneous brain activity (Zhao et al., 2020). Previous studies have shown that STG plays an important role in the recognition of actions and facial expressions (Allison et al., 2000; Manfredi et al., 2017). Furthermore, under neutral emotional activation, the latency of P170 in the left frontal lobe of yang personality is longer (Du et al., 2021). This implies that individuals with higher TYa scores have slower facial perception encoding speeds, potentially demonstrating a more thoughtful and detailed cognitive process in recognizing and processing emotional faces. These findings suggest that the TYa trait may be related to the brain’s differences in the processing of positive and negative emotional faces. However, it is important to highlight that no current research compares fMRI results for different personality traits under facial stimuli to demonstrate differences in brain region activation.

Additionally, the accuracy and F1 Score of classifiers built on different types of eye movement features vary across personality traits. Although we cannot conclusively identify the most predictive eye movement feature, the results of this study indicate that combining the three feature types leads to higher accuracy across most personality traits. While certain eye movement features provide valuable information for predicting specific personality traits, they may fail to capture the information needed to accurately predict others. This raises an important question about the relationship between eye movement features and personality traits. When observing emotional faces, complex eye movement patterns, eye-movement Shannon entropy and fixation duration of specific area of interest represent different eye movement behavior indicators, which provide various perspectives on the understanding of participants’ cognitive processing and attention distribution. Complex eye movement patterns provide information about overall and local eye movement behaviors, revealing participants’ processing of emotional faces in terms of both holistic and specific features (Holland and Komogortsev, 2013; Li et al., 2018). Eye-movement Shannon entropy reflects the expansiveness or concentration of attention distribution, providing insights into participants’ visual attention distribution when observing emotional faces (Shiferaw et al., 2019; Wang et al., 2020). Fixation duration of specific area of interest reveals the participants’ attention to different parts of emotional faces, potentially uncovering the focus of participants’ attention on emotional facial features (Ma et al., 2022; Chung et al., 2023). The combined application of these three feature types can more comprehensively reflect the eye movement behavior of participants when observing emotional faces. Moreover, the results of this study indicate that the predictive performance of combining the three feature types is superior.

It is of great practical significance to accurately predict the five-pattern personality traits, because different yin and yang contents show different personality traits, and then show differences in metabolic function, body state and susceptible diseases. These personality traits may be related to specific disease tendencies. For example, some studies have pointed out that high scores of SYi and TYi may be related to diseases such as bipolar disorder (Li et al., 2021), generalized anxiety disorder and depression (Zheng et al., 2007; Cui et al., 2018), and people with high SYa scores may be more prone to hypertension (Liu et al., 2014). Our eye movement prediction model aids in early identification of the five-pattern personality traits, thus promoting early prevention and health maintenance and helping individuals avoid potential physical and mental health problems.

Numerous studies have shown similarities between the results of the five-pattern personality inventory and those of international personality scales. For example, TYa and SYa scores positively correlate with the extraversion dimension in Eysenck’s personality theory (Wang et al., 2019). A significant correspondence has been found between Cattell’s 16 personality traits and the five-pattern personality traits among university students (Han et al., 2023). Additionally, research on Traditional Chinese Medicine graduate students has found a notable correlation between the five-pattern personality traits and the Minnesota Multiphasic Personality Inventory, with each dimension associated with specific disease tendencies (Wang et al., 2012). These cross-cultural research findings not only validate the scientific basis and practicality of the five-pattern personality traits but also lay the foundation for its application across diverse populations.

Moreover, the eye tracker used in this study is a commercially available device with broad applicability. The five-pattern personality inventory is based on sufficient research, and its content and model have been validated and applied in many situations. At the same time, all the image stimuli used are pre-existing, which ensures the repeatability and accuracy of the experiment. In addition, feature extraction and classification algorithms are widely used machine learning tools, which have been deployed in many applications, not specific to physiological signal processing or personality detection. They hold potential for use in a wide range of scientific research and application practice in the future.

In the future, we will conduct further studies to validate the effect of using eye tracking to predict five-pattern personality traits across different demographics, including gender, age, education level, occupation, and clinical patients. Another area of study to focus on is identifying how different scenes and types of stimuli affect personality detection. In addition, the contribution of various eye movement features to personality detection needs further study to better understand the neuropsychological relationship between features and personality.

## Data Availability

The raw data supporting the conclusions of this article will be made available by the authors, without undue reservation.
